# Thirty years from now: future physics contributions in nuclear medicine

**DOI:** 10.1186/2197-7364-1-4

**Published:** 2014-05-01

**Authors:** Dale L Bailey

**Affiliations:** School of Physics and Faculty of Health Sciences, University of Sydney, Sydney, 2006 Australia; Department of Nuclear Medicine, Royal North Shore Hospital, St. Leonards, NSW 2065 Australia

## Abstract

**Background:**

This paper is the first in a series of invited perspectives by pioneers of nuclear medicine imaging and physics. A medical physicist and a nuclear medicine physician each take a backward and a forward look at the contributions of physics to nuclear medicine. Here, we provide a forward look from the medical physicist’s perspective.

**Discussion:**

The author examines a number of developments in nuclear medicine and discusses the ways in which physics has contributed to these. Future developments are postulated in the context of an increasingly personalised approach to medical diagnostics and therapies.

**Conclusions:**

A skill set for the next generation of medical physicists in nuclear medicine is proposed in the context of the increasing complexity of ‘Molecular Imaging’ in the next three decades. The author sees a shift away from ‘traditional’ roles in instrumentation QA to more innovative approaches in understanding radiobiology and human disease.

## Background

### Looking back 30 years

It is ironic for me to be asked to comment on the future of physics applied to nuclear medicine 30 years hence, as this year celebrates my entry into the field as a young science graduate 30 years ago. During this time, I have often been asked whether, given my time again, I would change anything in terms of my selected path of study and subsequent career and I am always quick to respond with ‘No, not a thing’. Thirty years is just slightly longer than the common period used by historians to constitute a ‘generation’ , typically from the birth of a female child to her becoming a mother, currently accepted as being around 27 years (a figure that is probably on the rise in advanced economies), so we are speculating in this article about what the future has in store for the next generation. These individuals are currently our post-doctoral fellows and junior physicists.

When I entered the field 30 years ago, the gamma camera had just become mobile and could be taken to the patient's bedside. It had also become capable of acquiring images from 360° about the subject and subsequently reconstructing them to give tomographic sections using dedicated hardware array processors. Single-photon emission computed tomography (SPECT) was in its infancy. As for positron emission tomography (PET), that was a very expensive research tool for the neurosciences, plus a smattering of cardiology, that we were taught about but had little practical chance of coming in contact with, especially in Australia which lacked a cyclotron for producing the required radionuclides to fuel the PET camera. There were, however, many PET systems being developed in North America and Europe, including a number of innovative designs which included time-of-flight capability. In fact, a PET image of the dopaminergic system was featured on the cover of *Nature* in 1983 [1]. In Australia, Nuclear Medicine was still in transition from its endocrinology origins to a fully fledged independent specialty of internal medicine. Radionuclide therapies were common, using ^131^I (as to this day) as well as ^32^P and ^85^Sr, unlike computed tomography (CT) scanners which were limited to larger teaching hospitals, or MRI which was yet to be comprehensively rolled out clinically. The first physicist in nuclear medicine that I worked with was an expert in measuring *in vivo* metabolism and sequestration of radiotracers, and we made very precise measurements of phenomena such as iron metabolism (with ^59^Fe) using a combination of a gamma probe counter and blood samples. Compared to this, it seemed to me that the ‘imaging’ part of nuclear medicine was its Achilles' heel; definitely this was the era of ‘unclear medicine’. Real nuclear medicine, for me, was in the wonderful measurements that followed the time course of a radiotracer *in vivo* and which we plotted by hand on graph paper and ‘fitted’ with a bendable ‘snake’ to draw a line-of-best-fit through the data points.

By the mid-1980s mini-computers were starting to appear in nuclear medicine departments interfaced to the gamma cameras. These were physically large (in footprint) such as the Digital Equipment Corporation's PDP-11 (Maynard, MA, USA) with large removable hard disks of 1-MB capacity. In spite of the limitations of the instrumentation for imaging at the time, we commenced a programme to develop SPECT quantification believing this to be a highly desirable characteristic for a cross-sectional radionuclide imaging technology. This aim has recently been achieved and commercial SPECT systems, designed to be quantitative, are beginning to appear [2]. And to think, it has only taken around 30 years, or one generation! Interestingly, our ‘noble aim’ to develop quantitative measurements *in vivo* with SPECT serendipitously lead us into developing a form of simultaneous ‘hybrid imaging’ , meaning, acquiring multiple types of data (e.g. morphological and functional) in a single scanning session, which my medical colleagues at the time in the mid-1980s recognised as beneficial for adding crude anatomical localisation to SPECT. This resulted in novel applications such as in verifying the positioning of intra-arterial catheters for advanced chemotherapy treatments [3] and for imaging the distribution of aerosols of different sizes in the airways [4].

I would characterise key developments in instrumentation and physics in nuclear medicine by decades as being:

- 1980s: *Genesis* of tomography in clinical practice;
- 1990s: *Exploration* of tomography and multi-modal imaging; introduction of semi-quantitative analysis;
- 2000s: *Revolution* of tomographic hybrid imaging (PET/CT and SPECT/CT) and demise of 2D planar imaging - start of era of ‘New Clear Medicine’;
- 2010s: *Evolution* of quantitative 3D imaging on hybrid devices (SPECT/CT, PET/CT PET/MRI) and widespread introduction of targeted therapies;
- 2020s: *Expansion* of multi-parametric (tri-modal+ ?) analysis from multi-modality data sets in diagnostics and therapeutics - new interpretation criteria, understanding of radiobiology and individualised therapies;
- 2030s: *Integration* of genome-specific targeted diagnostics and therapies utilising *in vivo* whole-body assessment of tissue morphology, physiology and biochemistry and the micro-environment combined with complementary tissue and serum biomarkers to design super-selective therapies for every individual and monitor response to treatment in routine clinical practice.

**Figure 1:**
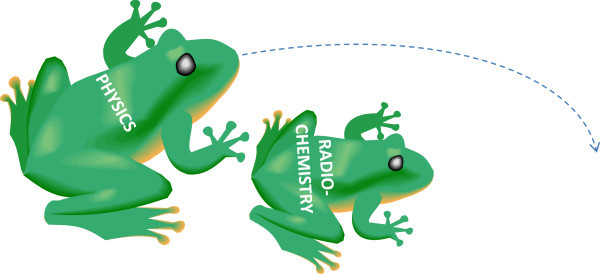
**Developments in physics and radiochemistry.** These developments often appear to occur at different times and ‘leap frog’ each other frequently, requiring the trailing discipline to ‘catch up’ with new developments to match the other's latest innovation.

It has often seemed to me that developments in the field do not progress together neatly in tandem, but rather that advances in radiochemistry and in physics are often out of synchrony with each other and may, in fact, drive each other forward almost appearing to ‘leap frog’ over each other (Figure 1). For example, a radiopharmaceutical was introduced in the 1990s to image prostate cancer using SPECT; however, while uptake in the positive lesions was high, there was also a lot of non-specific uptake in nearby structures which compromised image interpretation. The radiopharmaceutical experienced a re-birth, however, with the introduction of SPECT/CT, where all tissues exhibiting uptake could be readily identified. In this example, it could be argued that the introduction of the radiopharmaceutical was ahead of the appropriate technology with which to image it and that this was only corrected when new imaging devices were introduced. Today, perhaps, it might be suggested that the introduction of PET/MRI has occurred prior to the realisation of an imaging investigation for which it provides definitive and unique information [5]. Sometimes, radiochemistry developments get ahead of the instrumentation and physics required to exploit it to its full advantage, and sometimes, the instrumentation and algorithms get ahead of the available radiopharmaceuticals, as illustrated in Figure 1.

One useful case example to look at is the development of clinical PET scanning in the period from 1981 to 2011. The workhorse radiopharmaceutical for PET, [^18^F]-fluorodeoxyglucose (FDG), was already in use in the early 1980s [6]. The 1991 editorial article in the *Journal of Nuclear Medicine* by Henry Wagner screamed from the front cover *Clinical PET: Its Time Has Come*[7]. PET/CT was subsequently introduced by Townsend & Beyer et al. towards the end of the 1990s [8], and by 2011, PET/CT using FDG was one of the most significant clinical tests worldwide in staging, monitoring and managing patients with a variety of cancers. What had changed between 1981 and 1991, between 1991 and 2001, and between 2001 and 2011? We can start with what did not change; the types of cancer the patients had, and FDG. What did change is totally dominated by developments in physics and instrumentation:

- The introduction of ‘whole-body’ PET scanning for the application of cancer imaging by the UCLA group [9];
- The introduction of PET/CT which had a massive impact in a number of areas but particularly in providing rapid, high quality CT data for attenuation correction (thus greatly enhancing throughput) and anatomical localisation of FDG uptake in the PET scan; and,
- New scintillation crystals (i.e. LSO) to allow PET scanners to operate efficiently in 3D acquisition mode combined with the development of 3D reconstruction algorithms, firstly using classical filtered back projection [10, 11], but subsequently with optimised iterative algorithms such as the ordered subset expectation maximisation (OSEM) algorithm [12].

In addition, the questions being asked of the technique, moving from trying to estimate the degree of malignancy of the primary tumour on to staging the local nodal and distant metastatic spread of the cancer, were altered by the availability of the new imaging methodologies. This could be characterised as the development of the radiopharmaceutical being 10 or even 20 years ahead of its ultimate realisation as a clinical tool, thus demonstrating that the leap frog model does not always apply. However, it does demonstrate that the ability to obtain the vital, essential clinical information that we take as routine today required a series of developments on the instrumentation and image reconstruction side. In short, it was the developments in physics and instrumentation that brought PET from being a neuroscience research tool to being the imaging test of choice in staging and managing a large number of common malignancies. To all of the physicists and engineers who contributed to this transition, take a bow!

## Discussion

### Lessons learned

It is a long-standing taunt in our field that if you want to make a medical physicist anxious, you just ask him or her what it *is* that they actually do in their job. After being teased with this over many years, I offer the following response: we *measure* parameters in an attempt to *explain* natural phenomena. What tools do we require to do this? Leaving aside desirable personality characteristics such as enthusiasm, curiosity, insight and open-mindedness, which are all essential for good science, the greatest ‘gift’ I received was to be instructed during my university days and in my early career in software programming. I truly believe that the ability to write software code frees us to translate thoughts into practice, or, *in silico experimentation*. Hardware developments, at least in this field, tend to be expensive and on a much grander scale than can often be undertaken in the university physics laboratory or hospital department. But software development, as a form of *experimentation*, knows no such bounds. My own software skills could never be classified as ‘professional’; however, a large number of patient scans have been processed or analysed with novel software that I have written, and many of my publications are based on exploring software solutions to medical problems. One such example was developing a method to generate planar lung scan images (ventilation and perfusion scans) from SPECT data, a suggestion, or rather a challenge, set for me by one of my medical colleagues [13]. The solution proved to be a win-win situation for us and, as the results of numerous investigations have proven, for patients [14].

Moreover, to be able to work effectively with others, it is generally best if you share a common language. In the hospital medical physics setting, this language involves using medical terminology and requires the individual to have some rudimentary understanding of the anatomy, physiology and biology of not only the normal human condition but also the deranged state such as in cancer. How can we address the task of measuring, for example, liver function, if we do not understand the multiple inputs to the liver and the subsequent fate of a radiopharmaceutical after it is incorporated into the hepatic parenchyma? How can we judge the significance of a change in a PET FDG standardised uptake value (SUV) measurement if we do not understand the nature of errors, both random and systematic, arising from all sources including physiological, instrumental and algorithmic? And as medical treatment becomes increasingly personalised, the medical physicist should be aware of at least some of the impact that the genomic profile of an individual has in determining the true phenotyping of their condition and how this affects any imaging probe being used for diagnosis, staging or monitoring therapy response.

**Figure 2:**
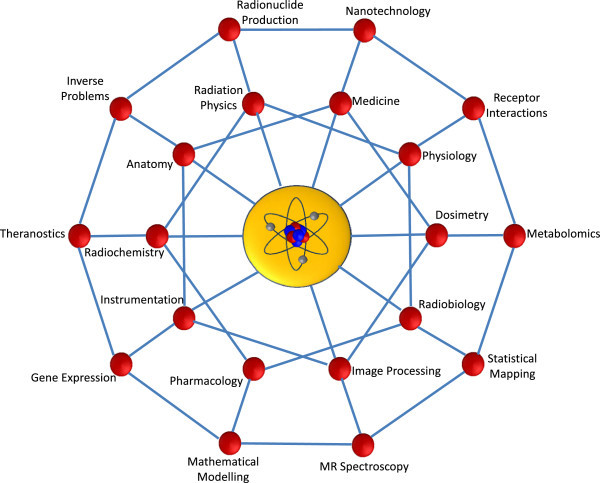
**The multiplicity of disciplines intersecting with nuclear medicine.** The practice of nuclear medicine includes multi-disciplinary interactions with numerous diverse areas, as illustrated in this schematic metaphor for ‘molecular imaging’ in the coming years with nuclear medicine at the core. Some of these interactions are well-established already, while others are emerging and will become more important for the next generation of medical physicists.

Finally, the field of nuclear medicine is enriched by the multiplicity of disciplines intersecting with it: e.g. physics, medicine, physiology, mathematical modelling, image processing, chemistry and pharmaceuticals, radiobiology, pharmacology, instrumentation, genetics, metabolomics, etc. (see Figure 2). Many of these interactions will necessarily involve collaborations with individuals from other disciplines, departments or institutions. Indeed, some of the most fruitful collaborations which I have been part of have been with individuals from outside my immediate group of colleagues. Therefore, participation beyond the immediate community of the local working environment (hospital, research group, university and industry) is strongly encouraged. It takes an investment of time but often reaps enormous rewards.

### Future directions in nuclear medicine

The 1980s saw the introduction of tomographic imaging in the clinic in the form of SPECT, and the subsequent decade followed up with the introduction of semi-quantitative objective analysis such as the development of scoring schemes referenced against normal databases for myocardial perfusion imaging (as well as improved radiopharmaceuticals for myocardial perfusion imaging labelled with Tc-99m with imaging properties better suited to SPECT, another example of the leap frog effect). We may well see a similar course of developments related to multi-modality imaging in the next decade, whereby different parameters from hybrid imaging devices (PET/CT, PET/MRI and SPECT/CT) are combined at the voxel level to produce a new ‘parametric image’ which combines joint information from the different modalities [15, 16]. This information is likely to be represented as a statistical estimate at a pre-determined level of significance, in much the same way that changes in cerebral function have been displayed within the framework of statistical parametric mapping, known as SPM [17], of functional MRI, PET or SPECT data for over 20 years.

Another area which is likely to attract considerable attention for the next generation of medical physicists in nuclear medicine is in better defining radionuclide therapy procedures based on a better understanding of the basic radiobiology of low flux rate, extended duration delivery of ionising radiation to biological targets. Much of today's understanding of radiobiology in human cancers is based on response to external beam radiation, which is delivered in short bursts over multiple irradiation sessions (fractions). This is very different to a radionuclide therapy which is more like a traditional brachytherapy in its mechanisms of slow, chronic delivery of ionising radiation.

Finally, after at least two generations essentially without change, the gamma camera is more than due for a total makeover. The gamma camera's main restrictions are related to its use of a physical, lead collimator to define the photon trajectories. This limits both sensitivity and spatial resolution. Alternatives, such as the Compton camera, exist but are still a long way from routine implementation. It is even likely that a Compton camera optimised for operating at energies around 0.5 MeV in single-photon mode could replace conventional coincidence detection in PET, as this mode of acquisition relies on the detection of both photons emitted in positron annihilation and represents a major restriction in sensitivity due to photon attenuation and scattering. Recall also that the branching ratio for the common positron emitters is close to 200%, i.e. there are two photons released for every decay, also giving rise to potentially excellent sensitivity.

## Summary

### The skill set for the next generation

While there is always a need for quality assurance (QA) of all instrumentation and software, in an increasing digital world the reliability and stability of imaging equipment are becoming far more robust. Therefore, I expect tasks related to QA and QC to become less taxing on the next generation of medical physicists than they might have been in the past. At the same time, it is also true that systems are becoming more complex, and the need for in-depth understanding of the accuracy and cross-calibrations of the various devices contributing to a clinical study requires greater attention. Thus, there is a shift from the medical physicist whose primary responsibility was to maintain the performance and accuracy of equipment to one who has insight into the processes being employed and who can troubleshoot and, if need be, improve the systems. This requires more understanding of the role of the equipment or devices in the context of the clinical setting than may have been required previously. An excellent recent example is the contribution of one medical physicist (MAL) as an author in defining the PERCIST criteria for evaluating FDG PET scan responses to treatment for solid tumours [18].

As I have mentioned before, generating software is an essential skill for the future generation of medical physicists for maximal productivity and to contribute to any team effort to develop better methodologies. But the ability to write software alone is not sufficient - the medical physicist needs to understand the image formation process so as to be able to interact with the data appropriately. This incorporates aspects of image processing and linear systems theory. A greater understanding of image reconstruction, an example of an inverse problem, is also highly desirable.

Finally, the next-generation medical physicist must be able to work as part of a collaborative team comprising individuals from a number of disciplines and be able to communicate effectively with them. This reiterates my earlier point about sharing a common language. The medical physicist in the nuclear medicine department of the future should be able to participate in discussions with surgeons, physiologists, internal medicine specialists, imaging specialists, radiopharmaceutical scientists, technologists, engineers, computer scientists, statisticians, spectroscopers and biologists all to a greater or lesser extent. With such a language, and with the right approach, the next generation of medical physicists can look forward to not only making significant contributions to future practice in medicine but also enjoying the rich experience and reward derived from doing so. I wish them well with some mild degree of envy.

## Authors’ information

Dale Bailey holds undergraduate and masters degrees in Applied Physics from the University of Technology, Sydney (UTS) and a PhD from the University of Surrey, UK. He is currently Principal Physicist in the Department of Nuclear Medicine, Royal North Shore Hospital, Sydney. He is also Professor in Medical Radiation Sciences, a Clinical Professor in the Sydney Medical School and Honorary Associate of the School of Physics at the University of Sydney. His main area of interest is in the development of quantitative techniques using imaging of *in vivo* radionuclide tracers. He has extensive experience in functional imaging using both single photon tomography (SPECT) and positron emission tomography (PET). Dale is a Fellow of both the Australasian College of Physical Scientists & Engineers in Medicine (ACPSEM) and the UK Institute of Physics & Engineering in Medicine (IPEM). He is a scientific affiliate of the Royal College of Physicians (London).
